# Molecular Advances on Phytases in Barley and Wheat

**DOI:** 10.3390/ijms20102459

**Published:** 2019-05-18

**Authors:** Claus Krogh Madsen, Henrik Brinch-Pedersen

**Affiliations:** Department of Molecular Biology and Genetics, Research Center Flakkebjerg, Aarhus University, 4200-Slagelse, Denmark; ClausKrogh.Madsen@mbg.au.dk

**Keywords:** phytase, wheat, barley, purple acid phosphatase phytase, PAPhy, mature grain phytase activity (MGPA)

## Abstract

Phytases are pro-nutritional enzymes that hydrolyze phytate and make associated nutrients, such as phosphorous, iron, and zinc, bioavailable. Single-stomached animals and humans depend on phytase supplied through the diet or the action of phytase on the food before ingestion. As a result, phytases—or lack thereof—have a profound impact on agricultural ecosystems, resource management, animal health, and public health. Wheat, barley and their Triticeae relatives make exceptionally good natural sources of phytase. This review highlights advances in the understanding of the molecular basis of the phytase activity in wheat and barley, which has taken place over the past decade. It is shown how the phytase activity in the mature grains of wheat and barley can be ascribed to the *PAPhy_a* gene, which exists as a single gene in barley and in two or three homeologous copies in tetra- and hexaploid wheat, respectively. It is discussed how understanding the function and regulation of *PAPhy_a* may support the development of improved wheat and barley with even higher phytase activity.

## 1. Introduction

Phytases (myo-inositol hexakisphosphate 3-,6- and 5-phosphohydrolase, EC 3.1.3.8, EC 3.1.3.26 and EC 3.1.3.72) are phosphatases that can initiate the stepwise hydrolysis of phytate (IP6, myoinositol-(1,2,3,4,5,6)-hexakisphosphate) and thereby provide phosphate (P), inositol phosphates, and inositol for a range of cellular activities [1]. In addition to purely scientific inquiries, phytase research has for many years been driven by the urgent need for improving utilization of phytate-phosphorus in diets for single-stomached animals, such as pigs and poultry, and to reduce the anti-nutritional effect of non-digested IP6 chelating micronutrients in the digestive tracts of humans and animals. As such, phytases can be regarded as tools for managing global phosphate resources and for alleviating human micro-nutrient deficiencies mainly in the developing world.

IP6 is the main storage form of phosphate in plants, typically amounting 2/3 of the total P content in the seed (Table 1). IP6 is a strong chelator and exists in the plant seeds as an insoluble mixed salt with cations called phytin. In cereals and many other plant seeds, phytin forms spherical crystalloid inclusions called globoids inside protein storage vacuoles. The globoids are the principal site of phosphorous (P), potassium (K) and magnesium (Mg) in the mature cereal grain but they also contain calcium (Ca), iron (Fe), zinc (Zn), copper (Cu), manganese (Mn), sodium (Na), sulfur (S), and protein [2,3].

Micronutrients are also chelated by phytate in food and feed, and hydrolysis is most wanted for improving micronutrient bioavailability. The anti-nutritional effect of phytate is in particular regarded as critical for Fe and Zn, where phytate is considered the single most important anti-nutritional compound for the bioavailability of these two micronutrients [7]. Iron deficiency is the primary cause of anemia and ranks among the most widespread nutrient deficiencies, estimated to affect 1.6 billion people worldwide [8]. Iron deficiency anemia has been linked to maternal and prenatal mortality, and to impairment of cognitive skills and physical activity [9]. For zinc, around 800,000 child deaths worldwide per year are attributable to Zn deficiency [10] because it significantly increases the risk of diarrhea, pneumonia, and malaria. Moreover, Zn deficiency has been linked to the morbidity and mortality of children younger than five [11].

Humans and single-stomached animals have insufficient phytase activity in their digestive tract unless it is provided by the diet. Unfortunately, major food and feed components like rice, maize and soybeans contribute with phytate but negligible phytase [4]. Because of the missing phytase activity, the phytate passes largely un-digested through the single-stomached animals’ digestive tract and enters the environment when their manure is spread on agricultural fields. Moreover, to ensure that farm animals get the phosphate needed, bio-available mined P is added to the feed. However, this strategy has become critical in many regions of the world where intense livestock production and spreading of manure with high levels of undigested phytate P on oversupplied agricultural soil leads to run-off of phosphorus to aquatic ecosystems. The resulting eutrophication is a severe environmental risk [12]. However, also from a resource perspective, inefficient utilization of plant phytate P is inappropriate. P is a non-renewable resource, essential for efficient agricultural production, and complete depletion of mined P will have unmanageable consequences for global food production [13].

## 2. Plant and Microbial Phytases

IP6 is resistant to most phosphatases whereas the lower inositol phosphates can be degraded by a wider range of phosphatases. Phosphatases that can initiate the dephosphorylation of IP6 are classified as phytases. So far, four classes of phytases have been identified: (1) Histidine acid phosphatase (HAP), (2) purple acid phosphatase (PAP), (3) cysteine phosphatase (CP) and (4) β-propeller phytase (BPP). Each phytase type has unique structural features due to their distinct catalytic apparatus that allows them to utilize phytate as a substrate in various environments [14]. 

For decades, applied phytase research was focusing mainly on microbial phytases and to our knowledge, all commercial phytases currently used for feed supplementation are microbial enzymes belonging to the HAP class [14]. Similarly, until recently, microbial HAP phytases were used exclusively for increasing plant seeds phytase activity through transgenesis. However, scientific achievements in recent years have led to a substantially increased knowledge based on the complements of phytases, in particular barley and wheat, and have demonstrated significant potentials of their phytases as highly stable and potent enzymes with potentials both in feed and food (see later). 

## 3. Mature Grain Phytase Activity

When hydrated, the mature seed tissues activate a battery of preformed hydrolytic enzymes that degrades the large internal pool of IP6 but also storage compounds like lipids, carbohydrates, and proteins. When ungerminated seeds are used as feedstuffs, this battery of enzymes constitutes all plant-derived hydrolytic activities. We refer to this as the first wave of activity and for phytase, it constitutes what is called the mature grain phytase activity (MGPA). In parallel with imbibition, the embryo synthesizes and secretes the plant hormone gibberellic acid. The aleurone and the scutellum layer of the embryo are thereby turned into secretory tissues where a wide range of hydrolytic enzymes are synthesized and secreted into the endosperm for degradation of cell walls, starch grains and storage proteins—the second wave of hydrolysis [2].

Cereals generally express phytases to assist in their IP6 metabolism but the MGPA varies several orders of magnitude. This is in strong contrast to the modest variation in total and proportional content of seed IP6 (Table1).

## 4. Classes of Phytases in Barley and Wheat

In wheat and barley, two types of phytases have been described, phytases belonging to the HAP class and phytases belonging to the PAP class of phosphatases [1]. The HAP phytases belong to the multiple inositol polyphosphate phosphatase (MINPP) group [15]. MINPP phytases have been reported to be expressed both during grain development, and thereby, potentially contribute to the MGPA, and during germination contributing to the second wave of phytate hydrolysis. The PAP phytases are represented by the TaPAPhy_a/bs from wheat and the HvPAPhy_a/bs from barley, respectively. Expression analysis showed that *PAPhy_a* genes are preferentially expressed during grain filling whereas the *PAPhy_b* genes are preferentially expressed during germination [16]. The Km value with phytate as a substrate for recombinant wheat MINPP rTaPhyIIa2 phytase and barley rHvPhyIIb phytase is around ten-fold higher than for the rTaPAPhy_a/b and rHvPAPhy_a/b PAP phytases, indicating PAPhys to be more potent phytases than the HAPhys (Table 2).

The contribution of HvPAPhy_a and HvPAPhy_b to the total MGPA in barley was recently evaluated using CRISPR/Cas and TALEN [18]. TALEN- and CRISPR/Cas9 were used for introducing targeted mutations in the promoter of the barley phytase gene *HvPAPhy_a*. Barley lines with substantial deletions in the *HvPAPhy_a* promoter and 5’CDS retained <5% normal MGPA. This confirms that the barley PAPhy_a enzyme is the main contributor to the MGPA and can be regarded as the main target for modulating MGPA.

## 5. Biochemical Properties and Storage of the PAPhys

Wheat and barley mainly store phytate in the protein storage vacuoles (PSVs) of the aleurone layer. PAPhy accumulated during grain filling is localized in the same organelles [16]. This suggests that some mechanism protects the phytate from hydrolysis during grain filling. The PSV’s are rapidly acidified in response to gibberellic acid as germination commences. A decrease from pH 6.6 to 5.9 was reported in the PSV’s of barley protoplasts incubated with 5 µM GA_3_ and the authors speculated that pH might play a crucial role in regulating vacuolar hydrolases [19]. Recombinant wheat phytases rTaPAPhy_a1 and rTaPAPhy_b1 showed pH optima of 5.5 and 5, respectively [16]. Optima of pH 5 and 6 were measured for seed purified barley phytases P1 (=HvPAPhy_b) and P2 (=HvPAPhy_a) respectively [20]. This shows that the PAPhy’s are most active when the PSV is in the acidified lytic, state and the higher pH optimum of preformed PAPhy_a may even be an adaptation, which enhances activity in the earliest stages of germination. Nevertheless, rTaPAPhy_a retained some activity up to pH 7.5 so pH regulation of the enzyme alone does not offer a satisfying explanation for the protection of phytate against premature hydrolysis. The second layer of pH-dependent protection is provided by the substrate’s organization into globoids, which provides some degree of water exclusion and steric hindrance [3]. A membrane surrounds the globoids and immuno-gold localization suggests that PAPhy is located outside this membrane [16,21]. The membrane, therefore, seems to provide an additional layer of protection, by physical separation. The temperature optimum of the recombinant wheat enzymes was 50 and 55 °C, respectively. The temperature curves showed a broad peak with 50% activity already at 30–35 °C but decreasing sharply around 60 °C. Optima of 55 and 45 °C were reported for the seed purified HvPAPhy_a and HvPAPhy_b respectively [20]. The kinetic parameters of recombinant wheat and barley phytases at 36 °C and pH 5 are summarized in Table 2. The corresponding values for *Aspergillus ficuum* phytase are given for comparison [17]. The values for the PAPhys are very similar.

## 6. PAPhy Genetics

**Figure 1:**
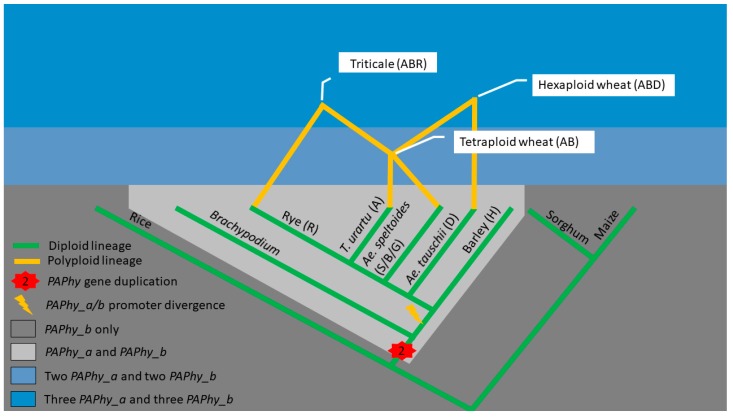
Key events in *PAPhy* evolution. Gene duplication and divergence of the PAPhy_a/b promoters and further duplications through polyploidization. For simplicity, rye is assumed to have just one *PAPhy_a* locus, and phylogenetic distance is not drawn to scale.

The *PAPhy_a* and *PAPhy_b* genes are paralogs, which originate from gene duplication in a common ancestor of wheat, barley, and rye (i.e., the Triticeae tribe) [22]. Rice, maize, and sorghum diverged from the Triticeae earlier and carry only one PAPhy gene whereas *Brachypodum distachyon* has the duplication but lack the conserved *PAPhy_a* promoter of the Triticeae (Figure 1) [22]. Allopolyploidzation has united the A and B genomes to form tetraploid wheat (e.g., durum wheat). *Triticum urartu* and *Aegilops speltoides* are the closest living relatives of the A and B genomes, respectively. Additional hybridizations have added the *Ae. tauschii* derived D genome to produce hexaploid wheat (bread wheat, spelt) and the rye derived R genome to produce triticale (Figure 1) [23]. Allopolyploidization results in large-scale gene duplication because most genes in one parent will have a homolog in the other parent species. In allopolyploids, such sets of genes are termed homeologs. Some homeologs may be lost or translocated as the polyploid species continues to evolve but the Triticeae *PAPhy* gene copy number and chromosomal localization are highly conserved. A single locus of *PAPhy_a* and *PAPhy_b* resides on chromosome 5 and 3, respectively in barley and on the homologous chromosomes on the three subgenomes of wheat [22]. The sequenced diploid members of the *Triticum* and *Aegilops*, i.e., *Ae. tauschii*, *Ae. speltoides*, *Ae. sharonensis*, *T. Urartu,* and *T. monococcum* also have one *PAPhy_a* and one *PAPhy_b* [24]. The chromosomal localization in these relatives has not been determined but it is reasonable to expect a conserved synteny between wheat and its ancestors *T. urartu*, *Ae. Speltoides,* and *Ae. tauschii*. *Secale* provides an exception since some members of this tribe have two *PAPhy_a* loci. In the case of domesticated rye, one and two *PAPhy_a* variants were isolated from the cultivars Imperial and Picasso, respectively [25]. Rye is an outbreeding species, unlike wheat and barley, and tends to have higher allele heterogeneity. Therefore, it cannot be excluded that the two variants from Picasso are alleles of the same gene even though phylogeny suggests that one allele may have been introgressed from *S. strictum* [25]. Thus, it is not known with certainty if domesticated rye has one or two *PAPhy_a* loci or whether it is cultivar-dependent.

**Figure 2:**
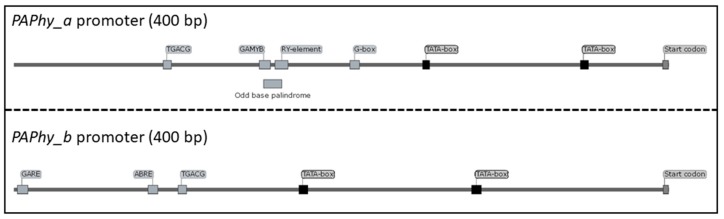
Consensus promoters of *PAPhy_a* and *PAPhy_b*, respectively, showing the most conserved cis-acting regulatory elements.

The intron/exon structure and the respective promoters of *PAPhy_a* and *PAPhy_b* are highly conserved. *PAPhy_a* has four introns and *PAPhy_b* has five. The position and, to a large extent, the size of the introns is conserved between Triticeae species and between the two paralogs [22]. Both genes have a core promoter of 3–400 base pairs which is conserved in all studied Triticeae. Both promoters have two TATA-boxes and upstream of those reside cis-acting regulatory elements consistent with the differential expression pattern of the two genes (Figure 2). For *PAPhy_b,* they are ABRE (abscisic-acid-responsive), TGACG (methyl-jasmonate-responsive) and—conserved in all examined *PAPhy_b* promoters—GARE (gibberellic acid responsive) [22]. The *PAPhy_a* promoter lacks these hormone responsive elements, except TGACG which is found at the very beginning of the conserved sequence. Instead, the most notable feature is a composite element with the consensus 5’ **GAACATG**AGT*CATG**CATG* 3’ which is made of the GAMYB binding motif (bold) [26], the odd base palindrome/GCN4 (underlined) [27,28] and the RY element (italic) [29]. These are all elements associated with seed development and storage proteins. Between the composite element and the first TATA box is a G-box motif [22]. Deletion of the odd base palindrome and the RY-element reduce barley MGPA by approximately 40% whereas deletion of the whole element and an additional 10 base pairs 3’ reduce MGPA by 75%. Deletions immediately 3’ of the composite element have even more severe effects [14]. It is not clear if this is caused by an unknown cis-acting regulatory element at that position or by the change in distance between the composite element and elements further downstream. 

## 7. Applied Potentials of the PAPhys

Although the PAPhy phytases appear to be somewhat less active than fungal HAP phytases, they certainly have technological potential. Transformation of barley with a genomic clone of *HvPAPhy_a,* including its native promoter (cisgenesis), more than doubled the MGPA [30]. Moreover, overexpressing *HvPAPhy_a* using the 35S promoter resulted in a line with impressive 40.000 FTU [31]. This level of MGPA represents more than a 20 fold increase and provides so much activity that <2% of the recombinant grains in a feed mixture could theoretically replace conventional phytase supplementation. Moreover, the enzyme activity was highly stable, mature leaves and straw accumulated PAPhy_a and high phytase activity remained after three years of storage. The PAPhy_a was easily extracted in water and could be added to feed or other processes.

The efficacy of a phytase in the digestive tract depends in part on how well intrinsic biochemical parameters, such as pH optimum, match the environment but also on resistance to proteolysis and inhibition. Feeding experiments are, therefore, necessary to prove efficacy. Early evidence for the efficacy of PAPhy_a was provided by comparing phosphorous and calcium utilization in pigs fed maize and triticale, respectively [32]. In this case, triticale-based feed performed better on both parameters, indicating an effect of the higher MGPA associated with triticale. In humans, an intervention study compared wheat bran with or without phytase (native vs. heat treated) in ileostomy patients. This resulted in recoveries of 40% vs. 95% phytate in the ileostomy content [33]. It is also possible to utilize the MGPA to achieve dephytination during food or feed preparation. Up to 96% of the phytate in whole grain wheat bread could be removed by simply adjusting the pH during proofing to five [34]. Similarly, fonio (*Digitaria exilis*) porridge could be dephytinized in one hour at 50 °C and pH 5 when 25% of wheat was included. This was demonstrated to significantly increase iron absorption in West African women [35]. Taken together, these studies demonstrate a lot of potential for the utilization of endogenous Triticeae PAPhy_a in food and feed. 

## 8. Achieving Higher MGPA with the PAPhys

Given the high conservation of the *PAPhy_a* coding sequence and highly similar enzymatic properties of all examined PAPhys, including PAPhy_b as well as rice and maize PAPhy, it seems unlikely that alternative *PAPhy_a* alleles, encoding decisively better enzymes, exist. On the other hand, increasing *PAPhy_a* expression has proven a viable strategy, as discussed. Efforts to increase MGPA by conventional breeding should, therefore, focus on the discovery of more highly expressed *PAPhy_a* alleles and the elimination of defective alleles.

Polyploids (e.g., wheat) are prone to acquire defective alleles because homeologs on the other subgenomes provide functional redundancy. Nevertheless, defective *PAPhy_a* alleles may reduce MGPA because the gene copies appear to function in an additive manner as demonstrated by cisgenic gene duplication [30]. Further support for this hypothesis is provided by the discovery of a defective *TaPAPhy_a2* gene in the wheat cultivars Skagen and Bob White that correlated with a lower MGPA compared to Chinese Spring, which has three normal loci [22,25]. A similar reduction of phytase activity has been observed in wheat lines with induced deleterious mutations in the *PAPhy_a* genes (author’s unpublished results). It is not known how commonly and how many different defective alleles exist in the elite wheat gene pool. A systematic investigation of this combined with the development of markers for the defective alleles would be helpful in ensuring a minimum MGPA in new cultivars.

More active alleles might be found in crop relatives. Wild emmer (*T. dicocoides*) can be crossed directly with other tetraploid wheat (e.g., durum wheat) and with hexaploid wheat trough synthetic hexaploids. Alleles from *Ae. tauscii* can also be introduced by this route or through the “octo-amphiploid bridge” [36]. A higher allele diversity is expected for these species because they have not gone through the bottlenecks of domestication and hexaploidization [37]. A small sampling of wheat and the closest relatives support this assumption [25]. Furthermore, it was found that at least one exotic allele had already been introduced in the elite wheat gene pool in the pursuit of other breeding goals—from *T. timopheevii*. In addition to the abovementioned, einkorn (wild and domesticated), *T. urartu*, rye, and *Ae. speltoides* could be used but here the introgression is more complicated.

Another strategy is mutagenesis. A wheat mutant with two to three times increased MGPA has been patented by the authors and co-workers [38]. This “HighPhy” wheat has an SNP in the composite element discussed above. Incremental replacement (1/3, 2/3, and 3/3) of conventional wheat with HighPhy all improved calcium and phosphorous digestibility compared to the control diet in a comprehensive feeding experiment with broiler chicken. Complete replacement with HighPhy wheat even significantly outperformed a control to which the standard dose of microbial phytase was added [39].

With the exception of the Triticeae cereals, none of the major food and feed grains accumulate nutritionally sufficient amounts of phytase during grain development [4,5]. Rather, phytase is synthesized de novo during germination in response to gibberellic acid. Introducing a transgene driven by a seed development specific promoter has, therefore, been considered the only realistic approach to introduce preformed phytase in major grains, such as maize, rice, and soybean. However, the recent advances in genome editing may change this because they enable targeted modification of endogenous genes [40]. In theory, this can introduce preformed phytase activity in one of two ways (a) by modifying the promoter of endogenous phytase genes to become active during grain development or (b) by modifying the active site of phosphatases already expressed during grain development so they become phytases. It seems possible to pursue option “a” by combining the lessons of the natural evolution of the *PAPhy_a* promoter from the *PAPhy_b* counterpart with target species-specific information on cis-acting regulatory elements involved in seed development-specific expression. The pursuit of option “b” would have to be guided by structural biology but has the advantage that mutations could be tested in a heterologous system before the more laborious plant gene editing. It is also likely that this approach would require smaller changes to the target gene sequence because a single base mutation is enough to change one critical amino acid. 

Proteinaceous inhibitors are known for many hydrolases including amylases, proteases, and xylanases. To our knowledge, such an inhibitor has never been reported for a phytase of microbial or plant origin. However, it was recently discovered that barley grain extracts do inhibit *Aspergillus* phytase. The inhibition could be reduced by the addition of pepstatin A. This suggests that the apparent inhibition is caused by an aspartic acid proteolytic activity [41]. It would represent a new and important variable, if plants use specialized proteases rather than conventional inhibitors to counteract phytases, e.g., from pathogens.

In conclusion, preformed wheat and barley phytase has the potential to counter the negative effects of phytate in the nutrition of single-stomached farm animals and humans alike. Realizing this potential depends on awareness because minor adjustments to processing may be needed. Generally, these adjustments should ensure that the phytase is not inactivated before ingestion or, alternatively, that the phytase has optimal conditions to perform the dephytination before inactivation. The higher the MGPA, the better the chances of a successful dephytination. Characterization of the *PAPhy_a* gene over the past decade should be most helpful for plant breeders who make MGPA a priority.

## Figures and Tables

**Table 1:** Total P, IP6 bound P, proportion of IP6 bound P, and phytase activity in wheat, barley and other cereals seeds. The number of samples is n and ± denotes the standard deviation. The range is given if *n* ≥ 2. Data from [4] ^1^, [5] ^2^, [6] ^3^.

Cereal	*n*	Total P (% of Dry Matter)	Total IP6 P (% of Dry Matter)	Percent IP6 out of Total P	Phytase Activity (FTU/kg) *
Wheat ^1^	13	0.33 ± 0.02	0.22 ± 0.02	67 ± 4.8	1193 ± 223
Wheat ^2^	18	0.40 ± 0.04	0.29 ± 0.04	73 ± 8.1	2886 ± 645
Wheat ^3^	30	0.29 ± 0.03	0.23 ± 0.03	79 ± 0.07	1637 ± 275
Barley ^1^	9	0.37 ± 0.02	0.22 ± 0.01	60 ± 2.4	582 ± 178
Barley ^2^	15	0.42 ± 0.4	0.26 ± 0.03	63 ± 3.5	2323 ± 648
Barley ^3^	21	0.31 ± 0.03	0.19 ± 0.02	61 ± 0.04	1016 ± 330
Rye ^1^	2	0.36 (0.35-0.36)	0.22 (0.20-0.23)	61 ± (56 -66)	5130 (4132-6127)
Rye ^2^	13	0.36 ± 0.02	0.24 ± 0.2	67 ± 5.0	6016 ± 1578
Rye ^3^	6	0.34 ± 0.03	0.20 ± 0.01	59 ± 0.02	5147 ± 649
Triticale ^1^	6	0.37 ± 0.02	0.25 ± 0.02	67 ± 3.7	1688 ± 227
Triticale ^2^	12	0.40 ± 0.03	0.28 ± 0.03	70 ± 5.4	2799 ± 501
Oats ^1^	6	0.36 ± 0.03	0.21 ± 0.04	59 ± 11	42 ± 50
Oats ^2^	6	0.37 ± 0.01	0.25 ± 0.02	67 ± 5.4	496 ± 35
Oats ^3^	9	0.29 ± 0.02	0.17 ± 0.03	59 ± 0.07	84 ± 39
Maize ^1^	11	0.28 ± 0.03	0.19 ± 0.03	68 ± 5.9	15 ± 18
Maize ^3^	7	0.32 ± 0.01	0.18 ± 0.01	78 ± 0.01	70 ± 7
Rice ^4^	1				72

* One FTU is the amount of enzyme that liberates 1 µmol of inorganic phosphorus per minute from sodium phytate at pH 5.5 and 37 °C.

**Table 2:** Kinetic parameters from HAP and PAPhy phytases from wheat, barley, and *Aspergillus ficuum*. Data from ^1^ [15], ^2^ [16], ^3^ [17].

Class	Enzyme	*K*m (µM)	*V*max (µmol/(min × mg))	*K*cat (s^−1^)	*K*cat/*K*m (s^−1^M^−1^)	pH Optimum
PAP Phytases	rTaPAPhy_a1 ^2^	35	223	279	796 × 10^4^	5.5
	rTaPAPhy_b1 ^2^	45	216	270	600 × 10^4^	5
	rHvPAPhy_a ^2^	36	208	260	722 × 10^4^	
	rHvPAPhy_b ^2^	46	202	253	550 × 10^4^	
HAP Phytases	rTaPhyIIa2 ^1^	246				4.5
	rHvPhyIIb ^1^	334				4.5
	*A. ficuum* phytase ^3^	27	-	348	129 × 10^5^	5.5
